# PBF, a Proto-oncogene in Esophageal Carcinoma

**DOI:** 10.1515/med-2019-0086

**Published:** 2019-10-13

**Authors:** Shi-hai Lian, Jun-ding Song, Yi Huang

**Affiliations:** 1 Department of Cardiothoracic surgery, Zaozhuang Municipal Hospital, 41# Longtou Rd, Zaozhuang 277100, Shandong Province, China; 2 Department of Cardiothoracic surgery, Zaozhuang Municipal Hospital, Zaozhuang 277000, Shandong Province, China

**Keywords:** PBF, proliferation, apoptosis, AKT/mTOR, Wnt3a/β-catenin

## Abstract

Emerging evidence shows that the pituitary tumour-transforming gene (PTTG)-binding factor (PBF) functions as a proto-oncogene in some tumors. However, the precise functions of PBF in tumorigenesis and its action mechanisms remain largely unknown. Here for the first time we demonstrated that PBF was associated with a tumor-related cell phenotype in esophageal carcinoma (ESCA) and identified the involved signaling pathways. PBF was up-regulated in ESCA tissues (Data from GEPIA) and cells. Then we down-regulated PBF in ESCA cell lines, Eca-109 and TE-1, by using RNAi technology. Cell function analysis suggested that down-regulation of PBF could inhibit tumor-related cell phenotypes, including proliferation, motility, apoptosis and cell cycle, in Eca-109 and TE-1 cells. Mechanism investigation suggested that apoptosis induced by PBF knockdown may be mediated by the activation of the mitochondrial apoptosis pathway and cell cycle arrest. AKT/mTOR and Wnt3a/β-catenin, key pathways in regulating tumor proliferation and metastasis, were found to be inactivated by the down-regulation of PBF in ESCA cells. In conclusion, our study demonstrates that PBF functions as a proto-oncogene in ESCA in vitro, which may be mediated through AKT/mTOR and Wnt3a/β-catenin pathways.

## 1 Introduction

Esophageal carcinoma (ESCA), with high invasiveness and lethality, is a type of malignant tumor occurring in the digestive system. At present, ESCA is the eighth most common cancer in the world and the sixth leading cause of cancer-related deaths [1]. ESCA is classified into esophageal adenocarcinoma (EAC or EAD) and esophageal squamous cell carcinoma (ESCC) according to its histological type, while ESCC accounts for 90% of cases of ESCA worldwide [1, 2]. Despite remarkable improvements in the treatment of ESCA in recent years, the current prognosis of ESCA is still poor because of the advanced diagnosis and recurrence after cancer treatment. The 5-year survival rate of patients is only 15%-25% [3, 4]. Therefore, it is very important to explore the pathogenesis of ESCC and find new therapeutic targets.

The pituitary tumour-transforming gene (PTTG)-binding factor (PBF), gaining its name due to directly interacting with PTTG1, is also known as PTTG1 interacting protein (PTTG1IP). The main role of PBF is to mediate the transfer of cytoplasmic PTTG1 into the nucleus, where it further regulates a series of biological processes, including metaphase-anaphase transition in mitosis, DNA repair and activation of gene transcription [5, 6, 7]. Due to the important roles in cellular physiological processes, PBF is found to be involved in the progression of different tumor types. For example, PBF overexpression is identified in thyroid, breast and colorectal carcinoma, and closely associated with the clinical features of patients, including tumor recurrence, metastasis and patients’ overall survival [8, 9, 10, 11]. PBF also transforms NIH 3T3 fibroblasts and induces tumors in nude mice. Moreover, transgenic thyroidal PBF in mice leads to hyperplasia and macrofollicular lesions by elevating AKT [9]. However, the function and regulating mechanisms of PBF in other carcinomas such as ESCA is still undefined.

In this study, using Eca-109 and TE-1 cell lines as a model, we first investigated the effect of PBF on the cell phenotype of ESCA *in vitro*, including proliferation, motility, apoptosis, cell cycle and so on. In addition, we also explored the regulating mechanisms of PBF in ESCA by focusing on AKT/mTOR and Wnt3a/β-catenin pathways.

## 2 Materials and methods

### 2.1 Cell lines and culture

Human normal esophageal epithelial cell line HEEC and ESCA cell lines Eca109, TE-1, EC9706, EC8712 and KYSE30 cells were purchased from the Cell Bank of the Chinese Academy of Sciences (Shanghai, China). Cells were maintained in DMEM medium plus 10% fetal bovine serum in humidified 5% CO_2_ at 37°C.

### 2.2 shRNA transfection and RT-qPCR detection for mRNA expression

The sh1-PBF (5’-GAGCTGCTTGTTCTCAGAA-3’), sh2-PBF (5’-CCTGTGAAGAGTGCCTGAA-3’) and sh3-PBF (5’-GAACGTCTCCGCCTGTTTA-3’) were synthesized by oligobio (China) and transfected into ESCA cells by using Lipofectamine 6000 (Beyotime, China). A scrambled shRNA was used as a negative control.

After transfection for 48 h, the total RNA of ESCA cells was collected by using TRIzol reagent (USA) and then reverse transcribed to cDNA using the Rever Tra Ace qPCR RT kit (TOYOBO, FSQ-101, Japan). The mRNA expression of PBF was examined by a FTC-3000 Real-Time Quantitative Thermal Cycler (Funglyn Biotech Inc, Canada). GAPDH was used as an internal control.

### 2.3 Cell proliferation analysis

CCK8 assay：ESCA cells transfected with shNC or sh2-PBF for 24 h were seeded in a 96-well plate at a density of 1×10^3^ per well. Cells in each well were incubated with 10 μl of CCK8 reagent (Solarbio science & technology, China) for 2 h at 24 h, 48 h and 72 h time point, respectively. Absorbance at 450 nm was detected by using an enzyme standard instrument.

Colony formation assay: After transfection for 24 h, ESCA cells (about 200) were seeded into a 6 cm dish with 5 ml of DMEM medium. Cells were normally cultured until forming large enough clones. After staining with 0.1% crystal violet, cell clones were imaged and counted under a microscope.

### 2.4 Cell migration and invasion analysis

Scratch assay: ESCA cells were transfected with shNC or sh2-PBF for 24 h and normally cultured until the cell monolayer was 95% confluent. Then a single-line wound was created using a pipette tip. After washing the detached cells with PBS, the ESCA cells were normally cultured in a serum-free medium for 24 h. The cell monolayer was imaged at 0 h and 24 h under a microscope and the wound closure (%) was calculated.

Transwell migration assay: The transfected ESCA cells (8×104) were resuspended in 500 μl of serum-free medium and were added in the transwell inserts (8 μm) (Corning, USA) placed in a 12-well plate. The lower chamber was added with 500 μl of complete medium as a nutrition inducer. Forty-eight hours later, cells that migrated through the membrane were fixed, stained and counted under a microscope. For the transwell invasion assay, a BioCoat Matrigel Invasion Chamber (BD Biosciences, USA) was used following the instructions.

### 2.5 Flow cytometry analysis for cell apoptosis and cycle

Apoptosis was detected using the Annexin V-FITC/PI Apoptosis detection Kit (4A Biotech. Ltd, Beijing, China) according to the manufacturer’s instructions. After transfection with shNC or sh2-PBF for 24 hours, ESCA cells were continually cultured in a serum-free medium for 24 h of starvation. Then the cells were collected and washed by cold PBS. Resuspend the cells by 1X binding buffer and adjust the cell density to 1-5 x 106/ml. Take 100 μl of cell suspension in a 5 ml flow tube, add 5 μl of Annexin V/FITC and incubate in the dark for 5 min. Add 10 μl of PI dye solution and 400 μl PBS for flow cytometry analysis. Flowjo software was used for processing results.

For the cell cycle, the transfected ESCA cells were digested with 0.25% trypsin, washed once with PBS and collected at a concentration of 1×106/ml. 1ml of cell suspension was centrifuged and incubated in 70% cold ethanol overnight at 4 °C. After being washed twice with PBS, cells were incubated with a pre-prepared 500 μL PI/RNase: A staining working solution at room temperature for 30-60 min in the dark. Then the cell cycle was analyzed using flow cytometry and Flowjo software.

### 2.6 Western blot

ESCA cells transfected with shNC or sh2-PBF were lysed by RIPA Lysis Buffer (CWBIO, China) for protein extraction. The concentration of proteins was determined using a BCA method. Then cell proteins were separated by a SDS-PAGE at 20 μg per lane and transferred onto a PVDF membrane. The membrane was then blocked, incubated with primary antibodies overnight at 4 °C. The primary antibodies, including anti-Bcl2 (1:500, ab59348, Abcam, United Kingdom), Bax (1:500, ab53154, Abcam, United Kingdom), cleaved-caspase 3 (1:200, ab2302, Abcam, United Kingdom), cleaved-caspase 9 (1:1000, ab2324, Abcam, United Kingdom), AKT (1:500, ab8805, Abcam, United Kingdom), CDK4 (1:500, ab137675, Abcam, United Kingdom), CDK6 (1:200, ab151247, Abcam, United Kingdom), cyclin D1 (1:500, ab226977, Abcam, United Kingdom), AKT (1:500, ab8805, Abcam, United Kingdom), p-AKT (phospho T308, 1:500, ab38449 ), mTOR (1:1000, ab2732, Abcam, United Kingdom), p-mTOR (phospho S2448, 1:1000, ab109268, Abcam, United Kingdom), p-p70S6K (phospho Thr389, 1:1000, #9205, CST, USA), Wnt3a (1:1000, ab28472, Abcam, United Kingdom), β-catenin (1:500, ab16051, Abcam, United Kingdom), E-cad (1:1000, ab40772, Abcam, United Kingdom), N-cad (1:1000, ab18203, Abcam, United Kingdom), Snail1 (1:500, 13099-1-AP, PTG, USA), Snail2 (1:1000, ab27568, Abcam, United Kingdom), Vimentin (1:1000, ab137321, Abcam, United Kingdom) and GAPDH (1:5000, 10494-1-AP, PTG, USA) were used in the present research. HRP sheep anti rabbit/mouse secondary antibodies (1:5000) were purchased from the PTG Company (USA). Protein bands were then developed by an ECL system and density-quantified by an ImageJ software.

### 2.7 Statistical analysis

All the experiments were performed three times. GraphPad Prism 7 was used for all statistical analysis. The comparisons between the two groups were analyzed using a student *t*-test. *P*<0.05 was considered statistically significant.

## 3 Results

### 3.1 PBF is up-regulated in ESCA but not correlated with patients’ survival

**Figure 1:**
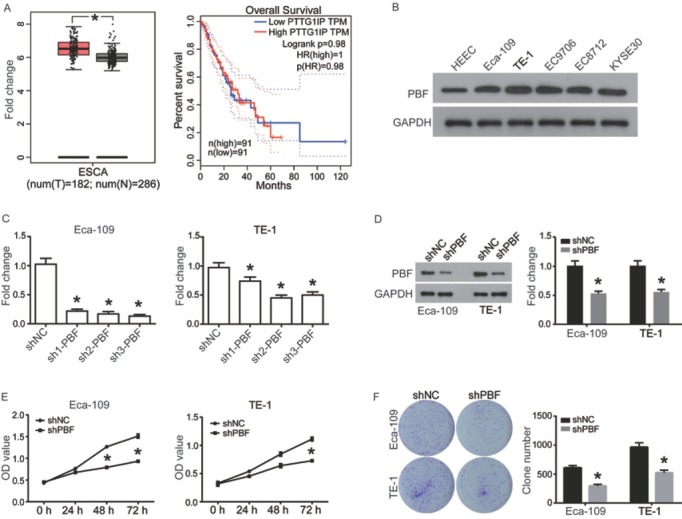
PBF was highly expressed in ESCA and down-regulation of PBF significantly inhibits proliferation of ESCAS cells. (A) Left: The expression of PBF in ESCA tissue (n=182) compared with normal controls (n=286); The red and gray boxes represent ESCA tissue and normal tissues, respectively; Right: The correlation of PBF mRNA expression with the overall survival of patients with ESCA; (B) PBF protein expression was investigated in human normal esophageal epithelial cell line HEEC and ESCA cell lines Eca109, TE-1, EC9706, EC8712 and KYSE30 cells by western blot analysis. (C)RT-qPCR was utilized for detecting mRNA expression of PBF in ESCA cells transfected with shNC or shPBFs for 24 h; (D) Western blot was used to confirm the interference effect of sh2-PBF on PBF protein level. (E) CCK8 assay was utilized for analyzing cell proliferation of Eca-109 and TE-1 cells, which were pre-transfected with shNC or sh2-PBF for 24 h; (F) Clone formation of the Eca-109 and TE-1 cells after transient transfection with shNC or sh2-PBF for 24 h. All experiments were repeated 3 times. *P represented significant difference.

Gene Expression Profiling Interactive Analysis (GEPIA) is a cancer-related RNA expression analysis website, data of which are from TCGA and GTEx databases [12]. On GEPIA we investigated the expression of PBF in ESCA and its correlation with patients’ survival. The results (Fig.1A Left) showed that PBF was significantly up-regulated in ESCA tissues (n=182) compared to normal controls (n=286, *P*<0.05). In addition, the survival curves from GEPIA analysis (Fig.1A Right) indicated that the correlation between PBF mRNA expression and the survival of ESCA patients isn’t significant (n_high_=91, n_low_=91, *P*>0.05). Moreover, we investigated the expression of PBF in a normal esophageal epithelial cell line HEEC and ESCA cell lines using western blot. The results indicated that PBF was highly expressed in Eca109, TE-1, EC9706, EC8712 and KYSE30 cells, compared with HEEC cells (Fig 1B). These data suggested that PBF might be involved in ESCA progression but didn’t affect the overall survival.

### 3.2 Down-regulation of PBF inhibits cell proliferation of ESCA

To evaluate the effect of PBF on cell phenotype of ESCA, Eca-109 and TE-1 cells were transfected with shRNA-PBF to establish PBF silenced ESCA cell models. The results in Fig.1C showed that all of 3 shRNAs could efficiently down-regulate the expression of PBF mRNA in ESCA cells, and sh2-PBF were selected due to the best interference efficiencies. The interference effect of sh2-PBF was also validated on the protein level by using western blot (Fig 1D). CCK8 assay showed that, compared to the negative control group (shNC), down-regulation of PBF led to a significantly inhibition on proliferation in both ESCA cell lines at 72 h time point (*P*<0.5, Fig. 1E). In addition, the colony formation ability of Eca-109 and TE-1 cells was also significantly inhibited by down-regulation of PBF compared to the shNC group (*P*<0.05, Fig.1F). Taken together, down-regulation of PBF induced growth inhibition on ESCA *in vitro*, suggesting that PBF might play an oncogenic role in ESCA.

### 3.3 Down-regulation of PBF inhibits cell mobility of ESCA

**Figure 2:**
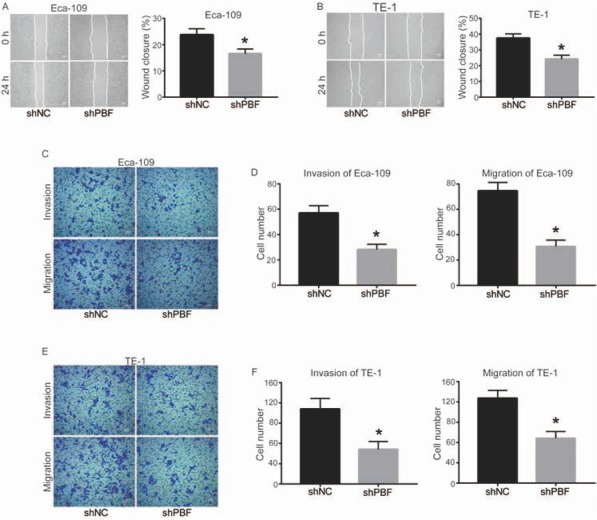
Down-regulation of PBF inhibits cell invasion and migration of ESCA. The ability of wound closure in (A) Eca-109 and (B) TE-1 cells was detected by scratch assay; bar = 1 mm (C) The images of invasive and migrated Eca-109 cells transfected by shNC or sh2-PBF for 48 h; bar = 100 μm. (D) Quantitative results of cell migration and invasion in Eca-109; (E) The image of invasive and migrated TE-1 cells transfected by shNC or sh2-PBF for 48 h; bar = 100 μm. (F) Quantitative results of cell migration and invasion in TE-1. All experiments were repeated 3 times. *P represented significant difference.

To determine whether down-regulation of PBF affects cell mobility of ESCA, scratch assay and transwell assay were performed. As shown in Fig.2A and B, compared to shNC group, wound closure (%) was significantly decreased by down-regulation of PBF in Eca-109 and TE-1 cells (*P*<0.05). Cell invasion and migration detected by transwell assay also suggested a significant inhibition when Eca-109 and TE-1 cells were transfected with shPBF (Fig 2C-F). Taken together, PBF functions as a promoter in ESCA cell migration and invasion, which is in accordance with the oncogenic role of PBF described above.

### 3.4 Down-regulation of PBF induces apoptosis and cell cycle arrest in ESCA

**Figure 3:**
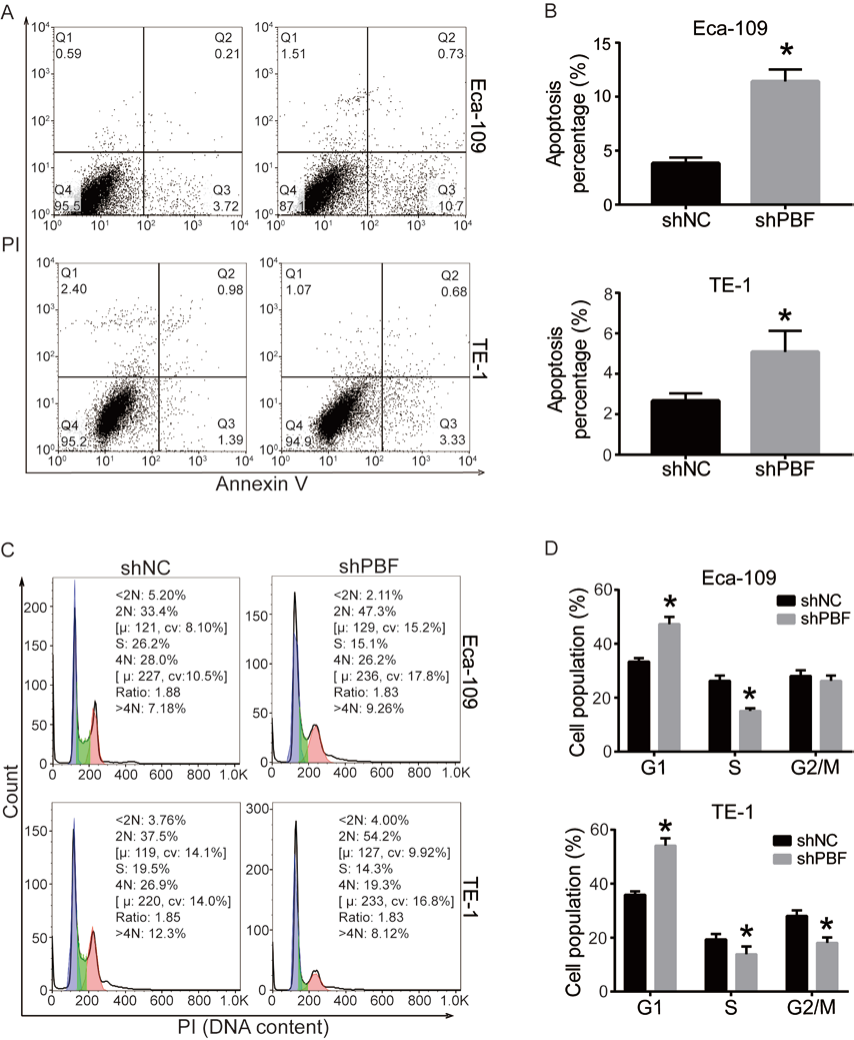
Down-regulation of PBF induces apoptosis and cell cycle arrest in ESCA. Cell apoptosis of Eca-109 and TE-1 was detected by flow cytometry, (A) the histogram of cell distribution after Annexin V/PI stainging, (B) quantitative results of apoptosis percentage. Cell cycle of shNC or sh2-PBF transfected Eca-109 and TE-1 cells was analyzed by PI-staining and flow cytometry, (C) cell distribution in cell cycle, (D) quantitative results of cell cycle distribution. All experiments were repeated 3 times. *P represented significant difference.

After observing a significant inhibition of proliferation and mobility by down-regulation of PBF, we further investigated the mechanisms contributing to this effect. We performed a flow cytometry analysis to determine the percentage of apoptotic cells (dyed by Annexin V/PI). Fig.3A exhibited a representative histogram of ESCA cells transfected with shPBF or shNC. Early Apoptotic cells were in the lower right quadrant (positive for Annexin V) and late apoptotic cells were in the upper right quadrant (positive for Annexin V/PI). Quantified results in Fig. 3B showed that the total apoptosis percentage of ESCA cells was significantly higher in the shPBF group than of that in the shNC group. The cell cycle was also analyzed by flow cytometry, in which ESCA cells were stained by PI to represent DNA content. As shown in Fig. 3C and D, ESCA cells distributed in G1 phase were significantly increased, while cells in S phase were decreased when PBF was down-regulated.

Taken together, down-regulation of PBF promotes cell apoptosis and induces cell cycle arrest in G1 phase in both Eca-109 and TE-1 cells.

### 3.5 Down-regulation of PBF activates mitochondrial pathway and Cyclin D1/CDK complex

**Figure 4:**
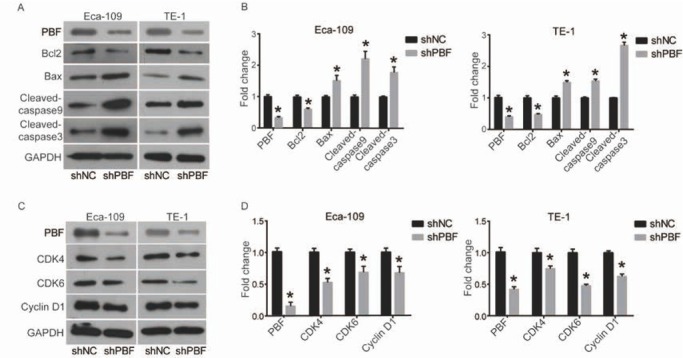
Down-regulation of PBF inactivates mitochondrial apoptosis pathway and repressed cell cycle regulators. (A) and (B) The members of Mitochondrial apoptosis pathways, including Bcl2, Bax, Cleaved-caspase9 and Cleaved-caspase3, were detected by western blot and quantified in density by ImageJ software; (C) and (D) Cell cycle regulators, including Cyclin D1, CDK4 and CDK6, were detected by western blot and quantified in density by ImageJ software. All experiments were repeated 3 times. *P represented significant difference.

In order to further determine the mechanism of the pro-apoptosis effect of PBF knockdown in ESCA cells, we investigated the status of apoptosis-related mitochondrial pathways, including Bcl2, Bax, cleaved-Caspase 9 and cleaved-Caspase 3, by using western blot. As shown in Fig.4A and B, down-regulation of PBF significantly increased the expression of pro-apoptotic proteins, Bax, cleaved-Caspase 9 and cleaved-Caspase 3, and decreased the anti-apoptotic protein Bcl2, suggesting that the mitochondrial pathway was activated. In addition, cell cycle-related proteins, including Cyclin D1 and CDK4/6, were also analyzed. As shown in Fig. 4C and D, Cyclin D1 and CDK4/6 were all down-regulated in ESCA cells transfected with shPBF, compared with shNC. Taken together, these data suggest that PBF knockdown promotes ESCA cell apoptosis through the activation of mitochondrial pathways and induces cell cycle arrest by repressing Cyclin D1 and CDK4/6.

### 3.6 PBF knockdown-induced cell phenotypic changes may be through inactivation of AKT/mTOR and Wnt3a/β-catenin pathways

**Figure 5:**
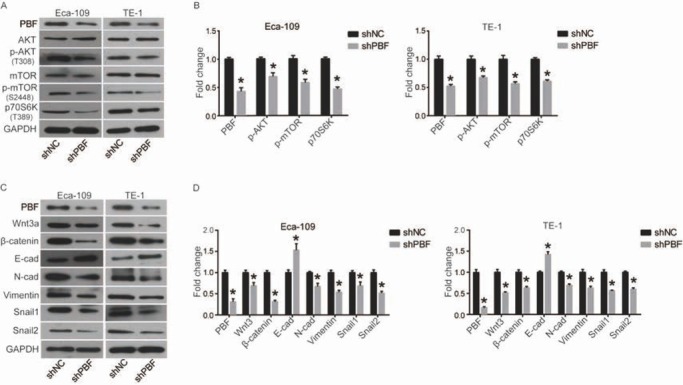
Down-regulation of PBF inhibits the AKT/mTOR and Wnt/β-catenin signaling pathways. (A) and (B) AKT/mTOR pathway including AKT, p-AKT, mTOR, p-mTOR and p-p70S6K was detected by western blot and quantified in density by ImageJ software; (C) and (D) Wnt/β-catenin pathway including Wnt3a, β-catenin, E-cad, N-cad, Vimentin, Snail1 and Snail2 was detected by western blot and quantified in density by ImageJ software. All experiments were repeated 3 times. *P represented significant difference.

Previous studies have discovered that the AKT/mTOR pathway plays a key role in the regulation of numerous biological processes, which could promote cell growth, survival and movements [13]. Moreover, activated mTOR will regulate a number of its downstream effectors important in cellular growth, such as p70S6 kinase (p70S6K), resulting in enhanced translation of a subset of genes that are required for protein synthesis and cell growth, inhibition of cell apoptosis, and acceleration of cell proliferation, which finally lead to a tumorigenesis [14, 15, 16]. Here we investigated the effect of PBF knockdown on AKT/mTOR pathway. As shown in Fig. 5A and B, p-AKT, p-mTOR and p70S6K were significantly decreased by down-regulation of PBF in Eca-109 and TE-1 cells, while the expression of total AKT and mTOR weren’t changed.

The Wnt/β-catenin pathway is reported to be involved in regulating the process of epithelial-mesenchymal transition (EMT), including decreasing cell adhesion and increasing cell migration and invasion [17, 18]. Here we found that down-regulation of PBF significantly inhibited the expression of Wnt3a and β-catenin (Fig.5C and D). The down-regulation of β-catenin resulted in the a changed expression of cell movement-associated proteins, including increased E-cad, decreased N-cad, Vimentin, Snail1 and Snail2, which indicated that EMT was activated (Fig. 5C and D).

Taken together, PBF knockdown-induced cell phenotypic changes, including inhibition of proliferation, mobility and so on, may be mediated by the inactivation of AKT/mTOR and Wnt3a/β-catenin pathways.

## 4 Discussion

PBF is a ubiquitous protein in human tissues and highly conserved in numerous species, suggesting that it plays an important role in the evolution of species [5, 19]. However, our understanding of the effect of PBF on cell functions in tumors remains relatively poor. Through bioinformatics analysis by GEPIA, we found that PBF mRNA was significantly up-regulated in ESCA tumor tissues compared with normal controls. Similarly, the upregulation of the PBF protein was also shown to be in most of the ESCA cell lines. The result was similar to the previous studies, reporting that PBF was overexpressed in thyroid, breast and colorectal carcinoma [10, 11, 20]. However, it showed no significant correlation between the expression level of PBF and the survival of patients with ESCA, which may be due to the complex tumor microenvironment. Previous study shows that PBF has the ability to induce malignant transformation of tumors [21]. For example, overexpression of PBF in thyroid could induce cell proliferation, and depletion of PBF will reduce cell viability of TPC1 cells in response to irradiation, which is mediated by regulating p53 activity [22]. Furthermore, PBF strongly promotes cellular migration and invasion in thyroid and breast cancer cells, and this activity is dependent on the expression of cortactin [23]. miR-584 inhibits the proliferation of glioma by down-regulation of PBF [24]. In this study, our data suggested that down-regulation of PBF inhibited ESCA cell proliferation, migration and invasion by inducing apoptosis and cell cycle arrest, which proved a proto-oncogenic role of PBF in ESCA *in vitro*.

In order to determine the action of mechanism of PBF in ESCA, we investigated the status of related signaling pathways by using western blot. Mitochondrial apoptotic pathway is one of the main pathways of apoptosis. Under stimulation of apoptotic signals, Bax translocates to the outer mitochondrial membrane and multimerize, forming membrane channels that stimulate mitochondria to release cytochrome C (Cyt C) [25, 26] . Released Cyt C is involved in forming apoptosis complex by interacting with Apaf-1 and Caspase 9, which further activates downstream Caspase 3 to induced apoptosis [25, 26]. Bcl-2 plays an anti-apoptotic role in this process mainly by inhibiting Bax [25]. Here we found that down-regulation of PBF induced apoptosis in ESCA by activating mitochondrial pathways, including increasing Bax, cleaved-Caspase 9 and cleaved-Caspase 3, while decreasing Bcl2. Cell cycle analysis indicated that PBF-induced cell cycle arrest was accompanied with suppressing Cyclin D1 and CDK4/6. Previous studies indicate that Cyclin D1 forms a complex with CDK4/6 to control G1-S transition in cell cycle, which can be downregulated by suppressing AKT signaling pathway [27] . Interestingly, our results showed the dysregulation of AKT pathway happened after PBF knockdown, which might be the reason why the expression of Cyclin D1 and CDK4/6 changed.

PBF could be associated with some pathways to exert a proto-oncogenic role in tumors. For example, it is found that overexpression of PBF in thyroid and colorectal carcinoma causes degradation of the tumor suppressor p53 by ubiquitination, leading to a p53 dysfunction related cell phenotype [10, 22]. The study of the murine transgenic model shows that overexpression of PBF could induce thyroid growth through up-regulating AKT, TSH receptor and downstream cyclin D1, all of which are important regulators of cell proliferation [9]. PBF also interacts with the sodium iodide symporter (NIS) and monocarboxylate transporter 8 (MCT8), modulating radioiodide uptake and thyroid hormone efflux, respectively [9]. In this study, we first found that down-the regulation of PBF inhibited AKT/mTOR and Wnt/β-catenin pathways, both of which might play important roles in tumor-related phenotypes.

In conclusion, our study suggested that PBF functions as a proto-oncogene in ESCA in vitro, which may be mediated through AKT/mTOR and Wnt3a/β-catenin pathways. Our finding will contribute to the therapy of ESCA in the future. However, our study also has some limitations. For example, PBF gain-of-function experiments in ESCA are not carried out in this study. Moreover, no rescue experiments are performed to further verify the oncogenic effects of PBF on ESCA cells via AKT/mTOR and Wnt/βcatenin signaling pathway indeed. These experiments will be investigated further in the following studies.
